# Strengthening and Targeted Rehabilitation for Optimal Neuromuscular Gains for chronic BACK pain (STRONG-BACK): protocol for a randomised controlled trial in participants with primary nociceptive pain drivers

**DOI:** 10.1136/bmjopen-2025-115538

**Published:** 2026-03-24

**Authors:** Maryse Fortin, Brent Rosenstein, Cleo Bertrand, Nicolas Vaillancourt, Alicia Wright, Chanelle Montpetit, Luciana Macedo, James Elliott, Chad E Cook, Yannick Tousignant-Laflamme, Jinhui Ma, M Gabrielle Pagé, Geoffrey Dover, Thien Thanh Dang-Vu, Michael H Weber

**Affiliations:** 1 Health, Kinesiology and Applied Physiology, Concordia University, Montreal, Quebec, Canada; 2 School of Health, Concordia University, Montreal, Quebec, Canada; 3 Centre de réadaptation Constance-Lethbridge, CRIR, Montreal, Quebec, Canada; 4 School of Rehabilitation Science, McMaster University, Hamilton, Ontario, Canada; 5 The University of Sydney Faculty of Medicine and Health, Sydney, New South Wales, Australia; 6 Northern Sydney Local Health District, St Leonards, New South Wales, Australia; 7 Department of Orthopedics, Duke University, Chapel Hill, North Carolina, USA; 8 School of Rehabilitation, Université de Sherbrooke, Sherbrooke, Quebec, Canada; 9 Université de Sherbrooke, Sherbrooke, Quebec, Canada; 10 Department of Health Research Methods, Evidence, and Impact, McMaster University, Hamilton, Ontario, Canada; 11 Anesthesiology and Pain Medicine, Université de Montréal, Montreal, Quebec, Canada; 12 CRCHUM, Montreal, Quebec, Canada; 13 Institut Universitaire de Gériatrie de Montreal, Montreal, Quebec, Canada; 14 McGill University Health Centre, Montreal, Quebec, Canada; 15 Orthopedic Surgery, University of Connecticut, Storrs, Connecticut, USA

**Keywords:** Back pain, PUBLIC HEALTH, REHABILITATION MEDICINE, RADIOLOGY & IMAGING

## Abstract

**Introduction:**

Exercise therapy is the most recommended treatment for chronic low back pain (LBP), with evidence supporting modest effects, likely due to the heterogeneity of patient presentations. Evidence suggests that matching individuals to the most appropriate exercise type could improve outcomes. Systematic reviews also emphasise that effective exercise interventions should be patient centred, target paraspinal muscle health and be of sufficient duration. This study addresses these gaps using a targeted care approach to identify a homogenous sample that is more likely to respond to our interventions. The inclusion of a sample with predominant nociceptive pain profile will be performed with the integration of the Pain and Disability Drivers Management Model (PDDM) and the Lumbar Spine Instability Questionnaire (LSIQ). The primary aim of this two-arm randomised controlled trial is to compare the effectiveness of motor control plus isolated lumbar extension exercises (MC+ILEX, arm 1) to free-weight resistance training (arm 2) in reducing LBP-related disability. Secondary aims include examining whether changes in multifidus composition mediate disability improvements comparing intervention effects on muscle size and quality, strength, mobility, pain, quality of life, sleep, physical activity and satisfaction; exploring baseline LSIQ scores and sex/gender as moderators of treatment response; and investigating participants’ perceptions and experiences of exercise therapy.

**Methods and analysis:**

A total of 106 participants will be recruited through primary and secondary care and randomised (1:1) to receive either MC+ILEX or free-weight resistance training. Both groups will complete 48 exercise sessions over 16 weeks. The primary outcome will be disability at 16 weeks, measured by the Oswestry Disability Index. Secondary outcomes include multifidus muscle composition and size, lumbar and gluteal muscle strength, hip range of motion, pain, physical and mental function, satisfaction and recovery, health-related quality of life, sleep quality and physical activity levels. Linear mixed-effects models will be used to assess primary and secondary outcomes. Regression analyses will explore whether baseline LSIQ scores moderate treatment effects on multifidus composition and other outcomes. A subsample of participants will undergo semistructured interviews before and after the intervention to explore their illness perceptions, illness mindsets, perceptions of exercise therapy, as well as their experiences and satisfaction with the two exercise interventions. Reflexive thematic analysis will be used to analyse qualitative data.

**Ethics and dissemination:**

This study received ethics approval from the Central Ethics Research Committee of the Quebec Minister of Health and Social Services (CCER-25-26-14). Results will be submitted to peer-reviewed journals and scientific meetings.

**Trial registration number:**

ISRCTN14864451.

**STRENGTHS AND LIMITATIONS OF THIS STUDY**

- The integration of the Pain and Disability Drivers Management Model and strict inclusion criteria will ensure inclusion of participants most likely to benefit from the exercise interventions.
- The use of advanced muscle imaging to directly link structural adaptations to meaningful functional outcomes.
- The qualitative strand will provide deeper insights into participants’ perceptions and experience of exercise therapy, guiding future research and improving intervention delivery.
- A limitation of this trial is the absence of blinding of the therapists and participants due to the nature of the exercise interventions.
- This trial will be conducted at a single research centre which may limit the generalisability of the findings to broader populations and clinical settings.

## Introduction

 Low back pain (LBP) remains the leading cause of disability,^1^ yet effective conservative management strategies are still limited despite decades of research.^2^ Exercise therapy is endorsed in clinical guidelines^3^ and supported by evidence arising from multiple systematic reviews^4^ as a first-line treatment of subacute and chronic non-specific LBP. Exercise is more effective than no intervention in managing chronic LBP^5^; however, the effect sizes for differences between exercise approaches remain modest.^4^ Several factors are likely to contribute to these limited effect sizes, including: (1) the heterogeneity of clinical presentations in LBP,^6^ (2) lack of risk-stratified approaches to enhance treatment selection,^7^ (3) suboptimal exercise dose prescription (dose/intensity are too low),^8^ (4) limited data regarding the influence of paraspinal muscle morphology on clinical outcomes^9^ and (5) clinicians holding unhelpful beliefs about LBP that may hinder appropriate exercise and reduce patient adherence.^10^ The proposed study aligns with international LBP initiatives^11^ and aims to address these gaps by implementing a rigorous clinical approach that integrates best practice recommendations while considering the complex biopsychosocial nature of LBP. An utmost limitation in most previous randomised controlled trials (RCTs) is the delivery of exercise without considering patient phenotype and/or the clinical heterogeneity of the chronic LBP population.^12^ This mismatch between interventions and patient profiles likely contributed to poorer outcomes and reduced effect sizes.^13^ To date, although promising multidimensional risk-stratification tools^14^ and biopsychosocial diagnostic frameworks ^15^ such as the Pain and Disability Drivers Management Model (PDDM)^16^ exist, few have been tested or implemented to guide exercise intervention RCTs.^10 16 17^

The International Association for the Study of Pain identified three dominant mechanisms of pain: nociceptive (eg, pain arising from tissue damage or inflammation), neuropathic (eg, nerve lesion) and nociplastic (eg, altered pain processing).^18^ Current evidence suggests that individuals living with chronic pain have overlapping mechanisms; however, it may be possible to identify dominant presentation. Selecting treatment based on these three pain mechanisms is increasingly recommended, as evidence suggests that they respond differently to interventions.^19^ Notably, patients with chronic LBP presenting with predominant nociplastic pain (eg, abnormal processing for nociceptive information)^20^ or substantial unhelpful beliefs such as fear of movement tend to respond poorly to specific exercise such as motor control (MC) and muscle strengthening.^12 21^ As a means to identify specific pain profiles across biopsychosocial domains, the PDDM—a theoretical framework—was operationalised through the development of a clinician-rated tool to facilitate the delivery of tailored intervention. The PDDM evaluates five biopsychosocial domains known to drive pain and disability in LBP: (1) nociceptive pain drivers, (2) nervous system dysfunction drivers, (3) comorbidity drivers, (4) cognitive-emotional drivers and (5) contextual drivers.^16 22^ This five-item rating scale builds on decades of research and has shown face and content validity,^23^ adequate inter-rater agreement^24^ and promising results to optimise LBP management in a recent pilot cluster non-RCT.^25^ In addition, empirical evidence supports the use of the Lumbar Spine Instability Questionnaire (LSIQ) (eg, a screening tool for clinical features associated with lumbar instability)^26^ as a predictive tool to identify patients most likely to benefit from MC and core exercise.^21^ Accordingly, this study will adopt a targeted care approach integrating both the PDDM and LSIQ to screen and include a homogenous sample of patients who are most likely to respond to our exercise interventions.

Patients with predominant nociceptive chronic LBP have structural changes in the paraspinal muscles, particularly the multifidus.^13 27^ Increased multifidus fatty infiltration is associated with higher pain severity, dysfunction, reduced strength, impaired voluntary contraction and poorer surgical outcomes.^28^,^30^ Fatty infiltration also reflects diminished muscle quality and metabolic resilience and is a recognised marker of sarcopenia.^31 32^ Accordingly, a large body of evidence supports the justification for considering paraspinal muscle dysfunction as a pillar component of rehabilitation programmes for LBP.^13^ Although exercises such as MC, isolated lumbar strengthening exercise and free-weight resistance training are promising forms of exercises for improving paraspinal muscle quality and function,^33^,^35^ most trials focused on patient-reported outcomes rather than structural muscle changes.^36^ Current recommendations from clinicians and evidence from systematic reviews reflect ongoing uncertainty about the specific mechanisms through which exercise improves pain and disability in chronic LBP. While it is widely believed that improving paraspinal muscle quality leads to better outcomes, this hypothesis remains largely unexplored, and a key question remains: *Does improvement in paraspinal muscle composition mediate changes in clinical outcomes in patients with predominant nociceptive chronic LBP?* Linking therapeutic outcomes to underlying mechanisms is essential to guide the clinical decision-making process. The approaches proposed in this study aim to address this gap, improve the effectiveness of commonly available interventions and advance precision rehabilitation.

It is increasingly acknowledged that the factors influencing participants’ engagement with interventions and resulting outcomes are more nuanced, dynamic and context specific than previously thought.^37 38^ For instance, mounting evidence suggests that patients’ illness perceptions, including beliefs about causes of LBP and perceived benefits of exercise, may have important impacts on treatment adherence and outcomes.^39 40^ Prior studies have noted discrepancies between quantitative measures and patient narratives,^41^ highlighting the need to investigate the complex phenomenon of LBP rehabilitation through multiple lenses.^42 43^ Careful consideration of participants’ subjective accounts is thus instrumental in moving towards context-sensitive, patient-centred care.^44^ Qualitative methods will be used to explore participants’ perceptions and experiences related to their LBP and to the two exercise interventions in the present trial. One-on-one semistructured interviews performed before and after completing the interventions will be used to collect rich data that will help shed light on potential mechanisms of treatment efficacy, identifying intervention aspects that may facilitate or interfere with the care provided.^45 46^ This line of inquiry is particularly relevant for programmes involving high doses of exercise over an extended period of time where interpersonal, motivational and environmental dimensions are expected to have important repercussions.

### Objectives

Our primary aim is to determine which 16-week exercise intervention, namely motor control plus isolated lumbar extension exercises (MC+ILEX, arm 1) or free-weight resistance training (arm 2), is more effective in improving disability in patients with predominant nociceptive chronic LBP. Secondary aims are to (1) assess whether changes in multifidus muscle composition mediate improvements in disability; (2) compare the effects of both interventions on multifidus size and quality, lumbar extensor and gluteal strength, hip range of motion (ROM), pain, health-related quality of life, sleep quality, physical activity level, and satisfaction and recovery; (3) explore whether baseline scores on the Lumbar Spinal Instability Questionnaire (LSIQ) and sex/gender act as moderators of treatment response at 16 weeks; and (4) explore participants’ perceptions, mindsets, experience and satisfaction of exercise therapy before and after completing the 16-week interventions.

## Methods and analysis

### Study design

**Figure 1:**
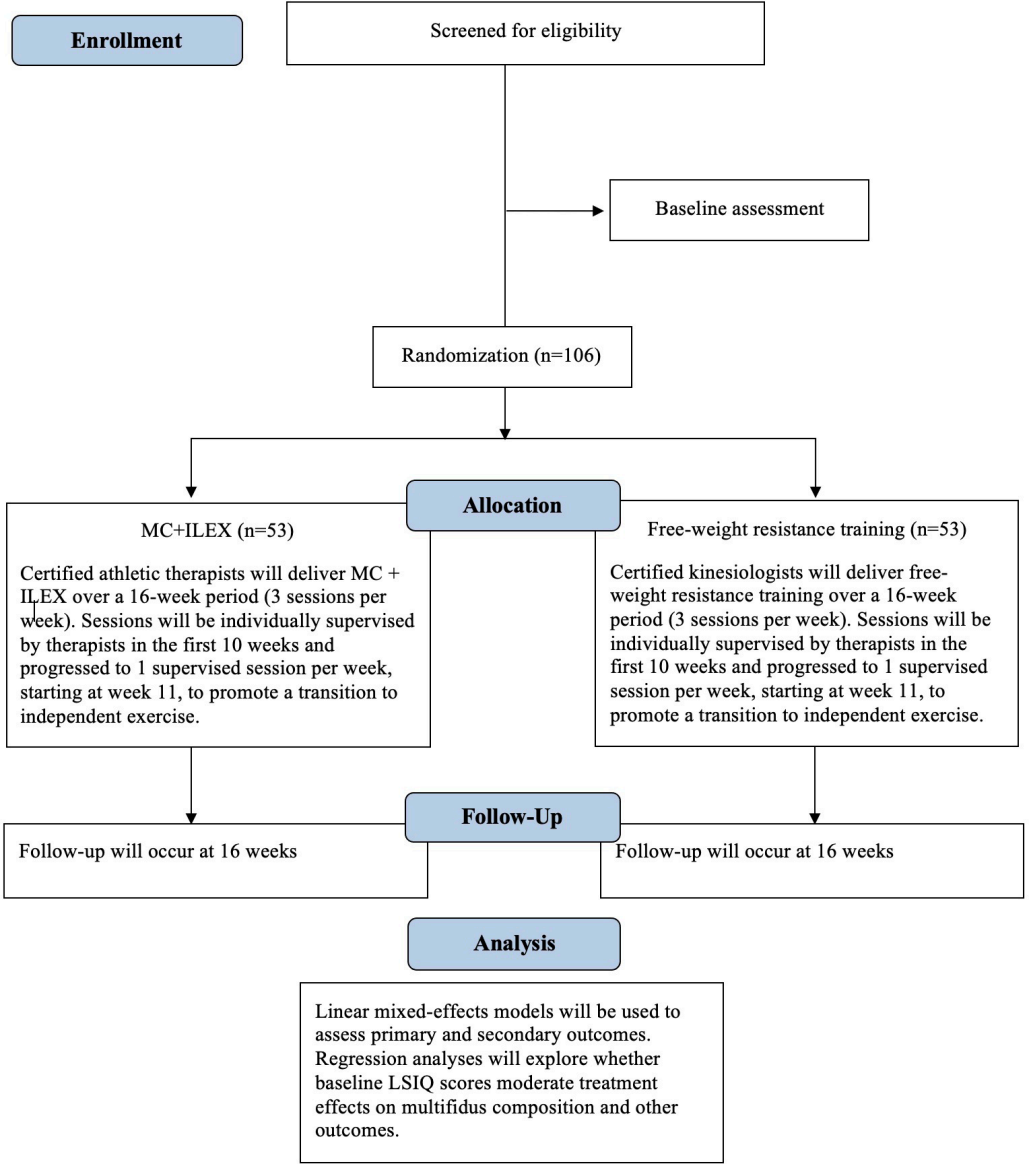
Flow diagram of the study process. LSIQ, Lumbar Spine Instability Questionnaire; MC+ILEX, motor control plus isolated lumbar extension exercise.

The proposed study is a two-arm RCT with assessor blinding (figure 1). This protocol was structured based on the Standard Protocol Items: Recommendations for Interventional Trials checklist for protocols of RCTs.^47^

### Participant recruitment

Participants irrespective of sex/gender will be recruited from primary care professionals (eg, physicians, physiotherapists, athletic therapists) working in the Montreal area. A primary care provider will receive study information and contact details to share with potential eligible patients, who can then contact the research team directly. Interested individuals will be followed up by a team member to discuss the study further, obtain consent (see consent form in the online supplemental material) and enrol participants. Additionally, recruitment will include community outreach through advertisements on social media and in public places.

### Participants

#### Inclusion criteria

Participants will be eligible for inclusion if they meet the following criteria:

- Chronic non-specific LBP (>3 months) defined based on the National Institutes of Health task force recommendations.^48^
- Between 18 and 60 years of age.
- Can speak English or French (to allow response to questionnaires and communication with therapist).
- Present with predominant nociceptive chronic LBP based on the PDDM, namely:

- Category A for *nociceptive drivers*.
- Category 0 for *nervous system dysfunction* (absence of neurological involvement).
- Category 0 for *comorbidity drivers* (absence of comorbidity).
- Category 0 for *cognitive-emotional factors*.
- Category 0 or A for *contextual drivers*.

#### Exclusion criteria

Participants will be excluded if they meet at least one of the following criteria:

- Previous spinal surgery or vertebral fractures.
- Radiculopathy.
- Other major lumbar spine structural abnormalities (eg, spondylolysis, spondylolisthesis or lumbar scoliosis >10°).
- Health conditions that would prevent active participation in exercise (eg, systemic inflammation).
- Institutional contraindications for undergoing an MRI exam (eg, pregnancy).
- Participation in high-intensity or progressive resistance training specifically targeting paraspinal muscle or trunk musculature ≥3 times per week within the past 8–12 weeks.

### Randomisation

Participants will be randomised into treatment groups using a computer-generated randomisation sequence prepared by a team member who is fully independent from all trial activities, including recruitment, assessment, treatment delivery and data analysis. The sequence will use block randomisation and will be stratified by baseline LSIQ scores (high >9; low ≤9). Allocation will be concealed using sequentially numbered, opaque, sealed envelopes assembled by the independent team member. At the baseline visit, the therapist will open the next envelope in sequence to determine group allocation, ensuring strict allocation concealment.

### Blinding

This trial will be randomised with concealed allocation and assessor blinding. True blinding of therapists and patients is not possible within an exercise trial.^49^ However, the therapists and participants will be blinded to the results of the LSIQ.

### Study interventions

Participants in each group will receive 48 sessions of approximately 1 hour over a 16-week period (three sessions per week). The treatment sessions will be individually supervised by certified athletic therapists or kinesiologists. Each participant will receive a free membership card to access the School of Health conditioning floor, where all research activities will be taking place.

#### MC+ILEX (experimental arm)

MC exercise was developed within a mechanical model of LBP. It is based on a large body of evidence suggesting that individuals with LBP have impaired control and coordination of the trunk muscles, especially the deep trunk muscles.^50^ The effectiveness of MC to improve pain and function in chronic LBP is supported by many systematic reviews.^4 50 51^ The MC exercise will be based on the programme reported by Hodges *et al*^52^ and similar to the protocol previously used in our pilot study.^53^ The intervention is based on assessment of the individual patient’s MC impairments and the patient’s individual treatment goals (set collaboratively with the therapist).

The first phase of the intervention involves assessment of the postures, muscle activation and breathing patterns. Based on the findings, the MC programme is designed to improve control of the trunk muscles through the increased activity and engagement of the deep trunk muscle (eg, multifidus and transverse abdominis) while minimising the reliance activity of any global muscle identified to be overactive, such as obliquus externus abdominis.^52^ Participants are taught how to contract the deep trunk muscles independently of the superficial trunk muscles and progress until they are able to maintain 10 repetitions with a 10-second hold of the target muscles,^54^ while maintaining normal respiration. During this stage, additional exercises for breathing control, posture of spine and lower limb and movement are performed. Once participants can activate their deep trunk muscles effectively (eg, with minimal compensation from superficial muscles while maintaining proper breathing), they will advance to phase 2. This phase aims to progressively increase the muscular load, transitioning from static to dynamic positions, all while preserving neutral lumbar alignment and ensuring coordinated activation of the deep trunk muscles. Exercise progression will be achieved by placing participants in increasingly challenging positions (eg, moving from supine to sitting), increasing the resistance through limb movements and incorporating dynamic stability elements such as unstable surfaces. The objective of this phase is to promote automatic activation of the deep trunk muscles and facilitate a coordinated engagement with the superficial muscles during functional tasks. Therefore, the final step of the intervention is progression to functional activities.

As done in our pilot work,^35^ MC will be combined with ILEX exercise to maximise the stimulus and activation of the lumbar extensors and induce adaptation.^55^ ILEX is typically performed with pelvic stabilisation to effectively target the lumbar extensors while minimising gluteal and hamstring activation.^56^ Research shows that ILEX training leads to clinically meaningful improvements in strength, pain and disability in individuals with chronic LBP,^55 56^ with strength gains often preceding hypertrophy due to neural adaptations.^57^ These findings,^58^ supported by our pilot data,^35^ justify the use of high-load training in the proposed trial to promote both neural and morphological adaptations.^59^ ILEX training will be performed on the MedX Lumbar Extension Machine (MedX, Ocala, Florida). This dynamometer can be used to assess isometric lumbar extension muscular strength (torque) in a seated position and accommodate dynamic resistance training through a full 72° ROM. During weeks 1–4, participants will be performing three sets of 12 repetitions at a resistance load of 60% of maximum recorded functional torque (one-repetition maximum, 1RM), and repetitions will be performed until momentary failure to control for intensity of effort.^57 60^ From week 5 to week 10, three sets of 10 repetitions will be performed at 80% of 1RM, and from week 11 to week 16, three sets of eight repetitions will be performed at 80% of 1RM, always to momentary failure. Participants will be instructed to perform repetitions taking at least 2 s to complete the concentric phase, hold the position/contraction for 1 s in full extension and then take 4 s to complete the eccentric phase.^60 61^ The resistance load will be increased by 5%^60 61^ at the next session once the participant is able to complete the set number of repetitions before failure.

#### Free-weight resistance training (control arm)

Free-weight resistance training, or compound exercise, is also among the most used forms of exercise therapy for chronic LBP, with systematic reviews documenting its effectiveness.^4 51^ The primary goal of resistance training in LBP is to address impairments in muscle strength, muscle quality and endurance.^57 58^ As previously mentioned, this intervention will be a replication of a previous study conducted by Welch *et al,*^34^ which reported a significant decrease in intramuscular fatty infiltration following a progressive 16-week free-weight resistance programme (without an ILEX component). The exercises will be delivered across two sessions. Session 1 will include the warm-up as prescribed by Welch *et al*,^34^ with minor regressions as needed (described below), followed by: isometric glute raise progressing to single-leg glute raise; mini squat to chair progressing to goblet squats; isometric split-squat hold progressing to split squats; planks on knees progressing to planks on toes; and neutral-grip machine row progressing to standing over-row. Session 2 will include a standardised warm-up (gluteus myofascial release; pelvis tilt as a regression for glute raise; multidirection weight transfer progressing to multidirection lunges; knee pulls followed by supine straight-leg raise as a regression for standing hamstring stretch; overhead squat; and assisted squat using long-loop resistance bands instead of the Smith machine), followed by: short hip hinge progressing to deadlift; step-ups; lat pulldown; side bridge on knees progressing to side bridge; and press-up on knees progressing to press-up on toes.^34^ Sessions will alternate weekly: two Session 1 and one Session 2 in 1 week, then one Session 1 and two Session 2 in the following week.^34^ During the first 4 weeks, participants will complete three sets of each exercise and the load will be set at 10RM. Starting at week 5, participants will complete three sets of five repetitions and the load will be set at 6–7RM. Participants who are unable to perform deadlifting off the floor (eg, limited lumbar flexion) will have the bar raised to a level in a squat rack, where a straight back can be maintained. Due to the need to learn new techniques/skills involving the coordination of movement and free weights (eg, force is not in a fixed plane like in a resistance machine),^62^ participants will be filmed when completing their exercises with coaching cues to maximise skill acquisition and learning.^63^

### Therapists

The therapists delivering both interventions will be trained by a psychologist in safety learning, therapeutic alliance and motivational interviewing to ensure consistent messaging, foster participant engagement and enhance overall quality of care.

### Data collection

All paraspinal muscle health-related outcomes will be collected in person at the School of Health (Concordia University). Self-reported questionnaire outcomes will occur preferentially using ‘*Manage My Pain*’ at appropriate intervals to the participants. Alternatively, if needed in French, self-reported measures may be answered over the phone or done in person using paper copies based on participants’ preference. Sleep quality will be collected at home using an actigraphy watch, as described below. All outcomes (primary and secondary) will be assessed at baseline and 16 weeks (end of the intervention) in both groups. Pain ratings will also be collected weekly throughout the entire intervention using a mobile app (refer to secondary outcomes). All the questionnaires will be administered via the clinically validated digital health application ‘*Manage My Pain’* by ManagingLife.^64^ Manage My Pain is a clinically validated app-based solution that helps people with chronic pain self-manage and better engage their care team. For clinicians and researchers, it facilitates a more effective way to measure outcomes along the patient journey.

### Baseline assessment

Sociodemographic characteristics will be acquired via the Canadian minimum dataset self-reported questionnaire at baseline, after randomisation.^65^

### Outcome measures

#### Primary outcome

##### Disability

##### Disability

The Oswestry Disability Index (ODI) will be used to measure participants’ level of self-reported disability in relation to LBP and common daily activities. This is a 10-item scale, which also includes pain severity outcomes, where each item is rated from 0 to 5 and higher scores are indicative of greater disability. This scale is the most commonly used to assess disability in LBP populations and has evidence of validity, reliability and responsiveness.^66^

### Secondary outcomes

#### Multifidus muscle composition

All participants will undergo a lumbosacral MRI evaluation using the School of Health’s (Concordia University) 3.0 Tesla GE scanner (Milwaukee, Wisconsin, USA). Body position will be standardised (eg, prone with knees flexed at 30°). Sagittal and axial T2-weighted (repetition time (TR): 3800, echo time (TE): 98) and axial 3D spoiled gradient-echo images using a 2-point Dixon fat-water separation technique (Lava-Flex, dual-echo acquisition; TE: 4.5, TE: minimum full, flip angle: 5) of the entire lumbar spine will be obtained for a total acquisition time of about 7 min. A standard phased-array body coil with 16 channels will be used, with 4 mm slice thickness, 180 mm^2^ field of view and 512×512 matrix. MRI slices will be acquired in a single block, and slice orientation will be standardized post-acquisition;^27^ coregistration will be used between time points. Multifidus muscle composition (eg, intramuscular fatty infiltration) will be assessed bilaterally at each lumbar level using the fat and water images, by calculating percent-fat signal fraction (%FSF) at each level using the following equation: %FSF=(Signalfat/[Signal water+SignalFat]×100). The %FSF will be measured automatically using convolutional neural networks (CNNs) with excellent reliability and validity, extensively studied by our research team.^67 68^

#### Multifidus muscle size

Multifidus cross-sectional area and cumulative 3D volume from MRI slices at each lumbar level (bilaterally) will be measured automatically using CNNs with excellent reliability and validity, extensively studied by our research team.^67 68^

#### Lumbar extensor muscle strength

Lumbar extensor muscle strength will be assessed with the use of the MedX Lumbar Extension Machine. The pelvis restraint system will be used to ensure isolation of the lumbar extensor muscles with the axis of movement being fixed between the vertebral levels of L5-S1. This dynamometry assesses isometric lumbar extension muscular strength (torque) in a seated position and accommodates the dynamic resistance through a full 72° ROM. Thus, maximum lumbar isometric contraction (Maximum voluntary isometric contraction, MVIC) in lumbar extensor muscle strength will be assessed in seven positions: 72°, 60°, 48°, 36°, 24°, 12° and 0° of flexion.^53 60 69^ Participants will first perform a controlled warm-up for approximately 1 min, and maximum strength testing will then begin.^69^ Verbal encouragements will be provided to encourage participants to generate maximum torque. The movement arm of the MedX machine is attached to a load cell that is interfaced with a computer recording the torque measurements.

#### Gluteal muscle strength

A handheld dynamometer VALD DynaMo (VALD Performance, Brisbane, Queensland, Australia) will be used to assess gluteal muscle strength. Participants will be placed on a therapy table in a prone position with their arms by their side to assess the gluteus maximus muscle strength with the knee at 90 degrees of flexion and the thigh/hip slightly extended (eg, off the table). Participants will be instructed to maintain this position for 5 s, creating an isometric contraction in the form of a ‘make’ test and asked to exert a maximal force against the handheld dynamometer. Measurements will be recorded in newton/torque. All participants will have a submaximal practice trial and then three measurements will be obtained on each side, with a break of 30–45 s in between each trial. The mean of the three measurements will be used in the analysis. Similarly, patients will be placed in a side-lying position with their arms by their side and with their top leg abducted, slightly extended and externally rotated to test the gluteus medius muscle. Again, participants will be instructed to maintain this position for 5 s, while exerting a maximal force against the handheld dynamometer. Three measurements will be acquired on each side. Handheld dynamometry has been shown to be a valid^70^ and highly reliable tool to assess gluteus muscle strength^71^ and has been recommended as a practical standard for clinical settings.

#### Hip ROM

Hip ROM and flexibility in flexion and extension will be assessed using the VALD DynaMo handheld dynamometer (VALD Performance), following standardised testing procedures. Each movement will be measured three times, and the average will be calculated. To minimise compensatory movements and ensure consistency, the hip will be stabilised using the ROM strap. For hip flexion, the participant will lie supine with both knees flexed at 90 degrees and will be asked to bring their knees to their chest. The VALD DynaMo will be attached to the lateral aspect and distal end of the test thigh. For hip extension, the participant will lie prone with both legs straight and will be instructed to lift their heels to the ceiling; the device will be fixated to the lateral side and distal thigh using the ROM strap. A handheld dynamometer VALD DynaMo will also be used to assess hip flexibility (flexion, extension). Hip flexibility will also be assessed using the DynaMo Plus. The straight leg raise test will evaluate hamstring flexibility, with the participant lying supine and the evaluator lifting the leg as high as possible while the device is attached to the distal lateral thigh. The modified Thomas test will assess hip flexor flexibility, with the participant seated at the edge of the table, grasping one knee to the chest and slowly lying supine on the table, while the opposite leg hangs freely over the edge of the table with the device attached to the lateral distal thigh using the ROM strap. The VALD DynaMo Plus has demonstrated excellent reliability for strength and ROM assessment, with intraclass correlation coefficients exceeding 0.90 in most tests.^72^

#### Impact of LBP: Patient-Reported Outcomes Measurement Information System

The Patient-Reported Outcomes Measurement Information System (PROMIS-29) v2.0 evaluates seven domains with four questions each, including: physical function, anxiety, depression, fatigue, sleep disturbance, ability to participate in social roles and activities, and pain interference and intensity.^73^ This questionnaire will be used to assess pain and how it interferes with physical and mental function.

#### Pain

We will monitor pain level with the Numerical Rating Scale (NPR) from 0 to 10 (where 0 is no pain and 10 is the most extreme pain) using the ‘*Manage My Pain’* app to assess weekly pain level. Both PROMIS-29 and NPR have excellent reliability and validity and can be used to detect clinically significant changes in perceived pain.^74 75^

#### Health-related quality of life

The 12-item Short Form Health Survey is the condensed form of the previous 36-item survey and will be used to assess participants’ health-related quality of life. The 12-item survey consists of eight domains that assess both physical and mental components of health. Scores from each of the 12 questions are combined to give an overall score between 0 and 100, with a score of 100 indicating the highest level of health. Given that this is a condensed version of a longer and established questionnaire, it has been extensively tested and shown to be both reliable and valid.^76^

#### Sleep quality

At both preintervention and postintervention, participants will be sent a wrist-worn actigraph (AX3, Axivity) in order to further evaluate sleep disturbance. Participants will be asked to use the actigraph for 7 days at home at both preintervention and postintervention for objective estimation of average sleep latency, wake after sleep onset, total sleep time and sleep efficiency, and will be asked to complete a sleep diary during the same period. While sleep disturbances are common in LBP, the effect of exercise on sleep quality remains to be established in this population.^77 78^

#### Level of physical activity

The International Physical Activity Questionnaire (IPAQ) will be used to measure participants’ level of physical activity. The IPAQ is a self-reported log of physical activity (metabolic equivalents (METs) based on intensity) in minutes per week over a period of 7 days. The level of physical activity is ranked either vigorous (8 METs), moderate (4 METs), walking (3.3 METs) and sitting/rest (1 MET) and must be allocated to the right category. The number of minutes per category is then combined, and the results are then organised as high, moderate or low physical activity based on the total MET minutes. The reliability and validity of this measure have been demonstrated.^79^

#### Satisfaction and Recovery Index level

The Satisfaction and Recovery Index (SRI) will be used to evaluate participants’ experience and perceived progress following the 16-week exercise intervention. The SRI is a validated patient-reported outcome measure assessing multiple dimensions of recovery, including satisfaction, perceived progress, symptom improvement and functional status.^80^ It consists of nine items rated on a 0–10 Likert scale, where higher scores indicate greater satisfaction and recovery. An importance-weighted composite score is calculated to reflect overall health-related satisfaction.

### Data monitoring

#### Adverse events

The occurrence of adverse events such as muscle soreness and transient increase in LBP levels will be monitored by the therapists throughout the intervention period and documented using open-ended questions during routine data collection follow-ups.

#### Adherence

Adherence to the exercise programme will be assessed through multiple methods. Therapists will maintain treatment records for each participant, which will be collected at the end of the study to evaluate attendance and engagement. Participants will also be asked to maintain a personal exercise log and will be provided with a training booklet that includes their individualised programme and wipe cards for tracking progress at the School of Health. Additionally, participants will wear an activity monitor (ActiGraph AX3) for seven consecutive days prior to the intervention and again at the 16-week follow-up to objectively measure step counts and intensity minutes, providing insight into overall physical activity levels.

#### Cointerventions

Participants will be asked to report any cointerventions (eg, chiropractic care, massage therapy) during each scheduled data collection follow-up. This will allow for the identification of potential crossovers. The use of pain medication will be permitted, as withholding it would be unethical; however, this information (type and dose) will be systematically recorded for both intervention groups.

### Qualitative strand

The qualitative strand is situated in a critical realist ontoepistemology,^81^ which treats knowledge and the individuals creating it as ‘contextually situated, partial, and always provisional’.^82^ Reflexive thematic analysis (RTA), as developed by Braun and Clarke,^83^ will be used within an experiential quality framework. That is, data collection and analysis are designed to capture and describe participants’ subjective perspectives.^83^ RTA is a theoretically flexible method for developing, analysing and interpreting patterns within qualitative datasets such as interview transcripts.^83^ Potential participants will be invited to take part in a one-on-one in-depth semistructured interview^84^ before starting the intervention and after having completed it. A purposive sampling strategy will be used to include individuals with varied demographic profiles, aiming for diversity of perspectives.^85^ Given the epistemological position of the qualitative strand, an exact sample size cannot be determined a priori.^86 87^ Rather, the concurrent collection and analysis of qualitative data will inform the number of participants included and orient purposive recruitment. It is provisionally estimated that 15–20 participants will be interviewed at each time point, based on prior studies with similar settings and methodologies.^88 89^ The concepts of information power^90^ and data adequacy^91^ will be used as guiding principles to determine and justify a final sample size. Semistructured interviews will take place via Zoom, a secure end-to-end encrypted video call platform.^92^ Two interview guides will be developed for the preintervention and postintervention interviews, listing questions and prompts meant to elicit information about perceptions and experiences of LBP, exercise beliefs and the present RCT. Each guide will be piloted with three participants at both time points to adjust and refine questions before continuing recruitment.^93^ Conversations will be audio recorded and transcribed verbatim and anonymised prior to data analysis. Interview transcripts will be analysed following the six phases of RTA outlined by Braun and Clarke^83^: (1) dataset familiarisation, (2) data coding, (3) initial theme generation, (4) theme development and review, (5) theme refining, defining and naming and (6) writing up. Coding will be both inductive and deductive; that is, the analysis will be grounded in participant narratives while being informed by theories including self-determination theory,^94^ social cognitive theory^95^ and the theory of planned behaviour.^96^ The following strategies will be employed to ensure methodological rigour: audit trail and reflexive journal, peer debriefs throughout data collection and analysis, and member reflections.^97^,^99^

### Sample size calculation

The sample size calculation was performed to compare the improvement in disability (eg, changes in ODI scores) from baseline to 16 weeks between MC+ILEX and free-weight resistance training exercise groups. This calculation was conducted using PASS 2023 software for two-sample t-test based on effect size. The minimum required sample size is 90 (45 per group) to detect a standardised effect size of 0.60 (Cohen’s d effect size), with 80% statistical power at the significance level of 0.05 (two-sided test), assuming an equal variance within the low (LSIQ<9) and high (LSIQ≥9) instability strata.^21^ Cohen’s d effect size of 0.60 falls within the range considered as a medium effect.^100^ This effect size is a conservative choice for the sample size calculation, given that the estimated Cohen’s d is approximately 0.79 based on the disability improvement observed in the MC+ILEX group from our previous work^35^ (mean=9.7, SD=10) and the disability improvement reported by Welch *et al*^34^ for the free-weight resistance training exercise group (mean=17.5, SD=5). A total of 106 participants (53 per group) will be recruited to account for a 15% loss to follow-up.^35 101^

### Statistical analysis

For the primary outcome, we will employ linear mixed-effects model for repeated measures over time using ODI as the dependent variable. The model will include effects for group (MC+ILEX vs free-weight resistance training), time (baseline vs 16 weeks) and group by time interaction. LSIQ score (high >9 and low ≤9) will be incorporated as a covariate and an unstructured covariance matrix will be used. Within the mixed model, we will calculate 95% CIs and p values for the two group contrasts, and for changes in ODI score within each group over 16 weeks. We will conduct similar analyses focusing on multifidus muscle composition and lumbar extensor muscle strength as separate dependent variables. To investigate if changes in multifidus muscle composition mediate improvements in disability, path analysis will be conducted to test the following paths: (a) group allocation at baseline (independent variable) → changes in multifidus muscle composition from baseline to 16 weeks (mediator); (b) changes in multifidus muscle composition from baseline to 16 weeks → changes in disability score (eg, ODI) from baseline to 16 weeks (dependent variable); and (c) group allocation at baseline → changes in disability score from baseline to 16 weeks. LSIQ score (high >9 and low ≤9) will be included in the model as an exogenous variable. For paths (b) and (c), disability score at baseline will be included as exogenous variables as well. The direct, indirect and total effects of treatment on the change in multifidus muscle composition and its 95% CI will be calculated based on non-parametric bootstrap. In addition, the mediation percentage is defined as the ratio of the indirect effect to the total effect, thereby quantifying the size of the mediation effect. The significance of the mediation effect will be tested using bootstrap sampling (times=1000). The model fit will be assessed using the following model fit indices: comparative fit index, Tucker-Lewis index and root mean square error of approximation.^102^ To explore if baseline score on the LSIQ can moderate the response to exercise training in chronic LBP at 16 weeks, we will conduct a linear regression model with changes in multifidus muscle composition from baseline to 16 weeks as the dependent variable. The independent variables will include group, LSIQ score (high >9 and low ≤9) and the interaction between group and LSIQ score (high >9 and low ≤9). Multifidus muscle composition at baseline will be adjusted as covariates in the model. Similar analyses will be conducted with changes in ODI score, multifidus size, lumbar extensor strength, pain, sleep and quality of life score from baseline to 16 weeks as the dependent variables, separately. Both complete case analysis and multiple imputation will be conducted to handle missing data. Any participant characteristics identified to be associated with missingness and the outcome will be integrated into the multiple imputation process. This will be accomplished using the fully conditional specification method fitted using predictive mean matching approach. Given the potential for missing data that are not missing at random, we will perform a sensitivity analysis to assess the robustness of our findings under the most unfavourable assumptions about the missing data. In this analysis, if we show that the MC+ILEX group is more effective than the free-weight resistance training exercise group in improving patients’ disability, all missing outcomes at 16 weeks will be imputed with the worst observed outcome in the MC+ILEX group and the best observed outcome in the free-weight resistance training exercise group, and vice versa.

### Data integrity

Data will be collected directly through the *ManagingLife* application. All data will be reviewed for completeness, and any inconsistencies will be investigated and corrected as needed. Deidentified data will be stored on a secure institutional managed server that complies with privacy standards. Access to the password-protected database will be restricted to authorised members of the study personnel.

### Retention of documents

Investigators will maintain complete and accurate research records to ensure full documentation of the study procedures and to support verification of study data. In accordance with institutional and federal research data retention requirements, all study data will be securely archived by Concordia University for a minimum period of 7 years.

### Patient and public involvement

The study protocol was developed in collaboration with clinicians and scientists with extensive experience in treating individuals with LBP. Patients with lived experience were also involved in the development of this study.

## Ethics and dissemination

### Ethics approval and trial registration

This study received ethics approval from the Central Ethics Research Committee of the Quebec Minister of Health and Social Services (CCER-25-26-14). The trial was registered prospectively (ISRCTN14864451) on ISRCTN study registry. Any important protocol modifications (eg, changes to eligibility criteria, outcomes or study procedures) will be submitted to the Research Ethics Board as a formal amendment prior to implementation. All coinvestigators and relevant study personnel will be notified of approved changes.

### Dissemination of findings

The principal investigator will present or publicise the results of this project in peer-reviewed journal publications and at national/international related conferences or meetings. The subject’s personal information will never be linked to any of the scientific data in question. On completion of the trial, and after publication of the manuscripts, data requests can be submitted to the primary investigator (MF).

### Timeline and feasibility

This project is expected to begin in winter 2026 and data collection should be completed within 2 years. We conducted a pilot trial at the School of Health (Concordia University) and were able to recruit our required sample of 50 participants within 4 months (eg, we had two recruitment periods of 2 months each). This was during the COVID-19 pandemic (2020–2021) while we had many additional hurdles and procedures in place. We expect to recruit 10 participants per month, which is in line with our pilot studies.^25 35 103^ We will recruit half of the required sample (53 patients) in the first year and the second half of the sample during the second year.

### Steering committee

The steering committee will consist of the principal investigator and coinvestigators, including two designated knowledge users. The committee will convene twice annually to review the study progress and provide oversight on study procedures. A data safety and monitoring committee will not be established, as adverse events are not a factor of concern in this trial.

## Discussion

This innovative and comprehensive RCT is designed to address multiple long-standing gaps in the exercise-based management of chronic LBP, offering a multifaceted approach that integrates clinical precision, mechanistic insight and patient-centred care. We will adopt a targeted care strategy by integrating the PDDM and LSIQ to screen and include a homogeneous sample of patients (eg, primary nociceptive pain) who are most likely to benefit from the exercise interventions, thereby advancing precision rehabilitation. This trial will also investigate whether improvements in muscle morphology mediate changes in disability, using state-of-the-art imaging and standardised measures of muscle composition. By linking therapeutic outcomes to underlying mechanisms, this study aims to inform and enhance clinical decision-making. Furthermore, the trial is designed to apply optimal exercise dose prescriptions based on previous evidence to produce meaningful improvements in paraspinal muscle quality and function.^34 35 53 104^ In addition to the exercise interventions, strategies will be implemented to enhance therapist confidence, foster effective communication, deliver consistent positive messaging and build a strong therapeutic alliance. Finally, a qualitative inquiry will be conducted to capture the depth and nuance of patient perspectives and satisfaction before and after the intervention, providing a comprehensive understanding of the patient experience and further informing clinical practice.

A limitation of this protocol is the lack of blinding for the therapists delivering and the participants undergoing MC+ILEX and free-weight resistance training due to the nature of the exercise interventions. However, this is a common and often unavoidable limitation of exercise-based interventions. Another limitation is that this trial will be conducted at a single research centre, which may affect the generalisability of the findings.

This trial will provide a unique opportunity to evaluate the effectiveness of MC+ILEX as compared with free-weight resistance training in a homogeneous sample of participants with primary nociceptive chronic LBP. The findings will provide robust scientific evidence to support a targeted LBP intervention. These results will have important implications for clinical practice, offering cost-effective and personalised treatment strategies. Furthermore, the study will contribute to the advancement of knowledge in musculoskeletal rehabilitation and inform future research directions in the field.

## Supplementary material

10.1136/bmjopen-2025-115538 online supplemental file 1
