# Supplementary material for: Strengthening and Targeted Rehabilitation for Optimal Neuromuscular Gains for chronic BACK pain (STRONG-BACK): protocol for a randomised controlled trial in participants with primary nociceptive pain drivers
Source: BMJ Open. 2026 Mar 24;16(3):e115538. doi: 10.1136/bmjopen-2025-115538 (PMC13034384; doi:10.1136/bmjopen-2025-115538)
Supplement: online supplemental file 1 [file bmjopen-16-3-s001.docx]

|  | **information form and consent** |
| --- | --- |
| **Title of research project:** | Strengthening and Targeting Rehabilitation for Optimal Neuromuscular Gains for chronic BACK pain (STRONG-BACK): A randomized controlled trial in participants with primary nociceptive pain drivers. |
| **Principal Investigator:** | Maryse Fortin, PhD, CAT(C), associate professor, Concordia University. |
| **Members of the research team:** | - Cleo Bertrand, PhD student & Research Coordinator, Concordia University - Chanelle Montpetit, PhD candidate, CAT(C), Concordia University - Nicolas Vaillancourt, MSc, PhD student, CAT(C), Concordia University - Alicia Wright, PhD student, Concordia University - Brent Rosenstein, MSc, PhD, Concordia University |
| **Funding organization :** | Canadian Institutes of Health Research (CIHR) |

**1. Introduction**

We are inviting you to participate in a research project. However, before agreeing to participate in this project and sign this information and consent form, please take the time to read, understand and carefully consider the following information.

This form may contain words that you do not understand. We invite you to ask any questions you may have to the research responsible for the research project or a member of his research staff and ask them to explain any word of information that is not clear.

**2. Nature and objectives of the research project**

The primary object of this research project is to evaluate the effect of a targeted exercise program versus general resistance exercise program on disability in individuals with chronic low back pain (LBP).

A secondary objective is to determine whether each intervention can improve back muscle health (size and composition) and function (strength), pain, quality of life, sleep quality, and physical activity levels in individuals with chronic LBP.

For the realization of this research project, we intend to recruit 158 subjects diagnosed with non-specific chronic LBP, i.e., pain of unspecified origin, male and female, aged between 18 and 60 years old.

**3. Research Project Process**

**3.1 Place of realization of the research project, duration and number of visits.**

This project will take place at the School of Health, Concordia University. Your participation in this research project will include a total of 48 visits, at a frequency of 3 visits per week, for a total duration of 16 weeks.

**3.2 Breakdown of groups**

By taking part in this research project, you will be assigned to one of the following groups:

- Group 1: Targeted exercise program
- Group 2: General exercise program

This research project is randomized, which means that you will be divided into one or the other of 2 groups. Your assignment to one or the other of these treatment groups is random, so you will not be able to choose your group. Thus, 1 in 2 (50%) will be included in group 1 and 1 in 2 (50%) will be included in group 2.

**3.3 Vigilance and habits**

We will ask you throughout the duration of your participation in this project to continue to follow your normal rhythm of life and usual physical activities.

**3.4 Common Activities – group 1 and group 2**

**3.4.1 First step: Evaluation before the start of the exercise program**

During your first visit, you will undergo a neurological screening. We will then ask you to complete some questionnaires regarding the intensity of your low back pain, and how your pain interferes with your daily life. We will also ask you to fill out a sociodemographic questionnaire. The completion of these questionnaires will take around 30 minutes. We will also introduce you to the application “Manage My Pain” by ManagingLife show you how to install it on your phone and how to use it.

Subsequently, in order to measure your back muscles’ morphology and function, we will ask you to undergo a:

- An MRI evaluation of your spine, glute muscles and hips for a duration time of around 45 minutes.
- A back and glute muscles strength test, for a duration time of around 30 minutes

**3.4.2. Second step: Exercise program and “Manage My Pain” application**

You will then begin a 16-week exercise program, with a frequency of 3 sessions a week. Each session will last between 45-60 minutes and will take place at the School of Health. A graduate exercise science student or athletic therapy student will supervise your exercise program. During the course of the intervention, you will be asked to use the “Manage My Pain” app to track your disability, pain, quality of life, sleep quality and physical activity levels. Pain ratings will be collected weekly throughout the program duration using the mobile app.

**3.4.3 Third step: Evaluation after the exercise program**

At the end of the 16-week program, in order for us to evaluate possible changes in your back musculature and symptoms, you will be requested to complete the same examination as performed during the first step and we will ask you to complete the same questionnaires.

**3.4.4 Fourth step: Interview**

Some of you will be invited to do an individual interview to ask you about your prior experiences with exercise for low back pain and expectations before the exercise program. In addition, we will ask you some questions regarding your experience and satisfaction after the exercise program.

These interviews will take place either in person or via a video conference and will last approximately 40 minutes.

The conversation will be recorded and transcribed for data analysis. Any identifiable information will be removed from the transcripts, and your data will be stored securely.

**3.5. Different Activities between the two groups**

For the duration of your participation in this project, the only difference between group 1 and group 2 will be the type of exercise you will receive. Your exercise program will include either targeted exercise for your back such as motor control and isolated lumbar extensor exercises or general muscle strengthening program.

All exercises of both groups will aim to improve disability and increase muscle function and strength.

**4. Description of the devices**

**4.1 Description of the magnetic resonance imaging (MRI) scanner**

In research, magnetic resonance imaging is used to provide imaging of the body and the brain, as well as their function.

For the realization of this research project, no substance will be injected. You will be lying on a mattress that will slide slowly in a large tube. The tube is open at both ends. An intercom system allows you to communicate with the medical imaging technologists as needed. For your convenience, we will ask you to wear either a headset, or protective earplugs to reduce the significant noise that is emitted by the device. While the unit is operating, it is important to stay still. To do this, a pad will be placed around your head to ensure immobility.

**5. Incidental findings**

Although they do not undergo a formal medical evaluation, the results of all tests, tasks and procedures that you have to do during your participation in this project can highlight problems previously unknown, called incidental findings. Therefore, in the presence of a particular feature, the researcher responsible for the project will call you to do a follow-up.

**6. Advantages associated with the research project**

You may gain personal benefits from your participation in this research project, but we cannot assure you. In addition, the results obtained will contribute to the advancement of scientific knowledge in this field of research.

**7. Disadvantages associated with the research project**

Besides the time required for your participation in this research project, there are no other disadvantages associated with your participation in this research project.

**8. Risk associated with the research project**

**8.1 MRI**

According to current knowledge, undergoing MRI examinations for the purpose of this research project will not put you at any risk, if you have no contraindications.

Due to the strength of the magnetic field emitted by the device, it is necessary to take certain precautions. This is why you must complete a detailed questionnaire in order to detect any contraindication, for example, the presence of a pacemaker, an aneurysm clip, a metal prosthesis, a prosthesis or cardiac valve clip, presence of metal in the eye or on the body, tattoo, piercing, dental braces or if you suffer from claustrophobia. The rigorous verification of the presence of contraindication will be assumed by the medical imaging technologist.

In addition, the conditions imposed by the use of the apparatus may result in discomfort from having to stand still and discomfort could also be associated with the noise which is generated by the operation of the apparatus. You might also feel some stress.

**8.2. Exercise Program**

It is possible that you feel some muscular stiffness after completing an exercise session, which is quite normal following a muscle strengthening program. The pain should disappear quickly in 1 or 2 days. All exercises will be adapted to your condition and physical level.

**9. Risks associated with pregnancy**

Participation in this research project may involve risks, known or unknown, for pregnant women, unborn children or breastfed infants. This is why pregnant or breastfeeding women cannot participate in this project.

Women likely to become pregnant will have to undergo a pregnancy test before the magnetic resonance imaging is performed and they will only be able to participate in this project if the result of the pregnancy test is negative.

**10. Voluntary participation and possibility of withdrawal**

Your participation in this research project is voluntary, and you are free to refuse to participate. You can also withdraw from the project at any time, without giving reasons, by telling the research team

The researcher in charge of the research project and Comité central d'éthique de la recherche du ministre de la santé et des services sociaux or the funding organization may terminate your participation, without your consent. This may happen if new findings or information indicates that your participation in the project is no longer in your best interest, if you do not follow the instructions of the research project or if there are administrative reasons to abandon the project.

If you withdraw or are removed from the project, the information and materiel already collected for this project will nevertheless conserved, analysed or used in order to ensure the integrity of the project.

Any new knowledge gained during the course of the project that could have an impact on your decision to continue to participate in this project will be communicated to you rapidly.

**11. Confidentiality**

During your participation in this project, the researcher responsible for the research project and the members of its research staff will gather your information in a research folder. Only the information necessary to meet the scientific objectives of the project will be collected.

This information may include information about your medical history related to your lower back pain, your lifestyle as well as the results of all tests, examinations and procedures that will be performed. Your file may also include other information such as your name, gender, date of birth and ethnicity.

All information collected will be kept confidential within the limits of the law. In order to preserve your identity and confidentiality of this information, you will only be identified by a code number. The key to the code linking your name to your research folder will be kept by the researcher responsible for this research project.

This research data will be kept for at least 7 years by the researcher responsible for this research project.

Research data may be published or be the subject of scientific discussion, but it will not be possible to identify you.

For surveillance, control, protection and security purposes, your research folder may be consulted by a person mandated by regulatory bodies as well as by representatives of the funding organization or the Comité central d'éthique de la recherche du ministre de la santé et des services sociaux. All these individuals and organizations adhere to a privacy policy.

You have the right to consult your research file to verify the information collected and have it rectified if necessary.

**12. Secondary use of research data**

Do you agree that your research data be used by the principal investigator to conduct other research projects?

These research projects will be evaluated and approved by the Ethics Committee of Concordia University prior to their implementation. In addition, the Research Ethics Board at Concordia University will be monitoring these studies.

Note that your research data will be securely stored by the researcher responsible for this research project on Concordia University’s computer servers. In order to preserve your identity and confidentiality of your research data, you will be identified only by a code number. The code key will be kept by the researcher responsible for this research project.

Your research data will be kept as long as is may be useful for the advancement of scientific knowledge. When they are no longer needed, your research data will be destroyed. In addition, note that at all times you may request the non-use of your research data by contacting the researcher responsible for the research project.

Do you agree that your research data be used in these conditions? **□ Yes □ No**

**13. Participation to subsequent studies**

Do you agree that the researcher in charge of the project or a member of his research staff resume contact with you to propose you to participate in other research projects? Of course, during this call, you will be free to accept or refuse to participate in the proposed research projects. **□ Yes □ No**

**14. In case of prejudice**

If you had suffered prejudice either due to your participation in the research project, you will receive all the care and services required by your state of health.

By agreeing to participate in this research project, you do not waive any of your rights nor release the researcher responsible for this research project, the funding organization and Concordia University for their civil and professional liability.

**15. Procedures in case of medical emergency**

Should a medical condition that would require immediate care, first aid will be provided by existing staff and arrangements will be made to transfer you, if necessary, at the emergency of a nearby hospital.

**16. Identification of resource persons**

If you have questions about the research or if you experience a problem that you believe is connected to your participation in the research project, you can contact the researcher in charge of the research project, Maryse Fortin, at the following number: (514) 848-2424 ext. 8642

For questions about your rights as a participant in this research project or if you have complaints or comments you can contact the Office of the Ombudsman Concordia University at the following number: (514) 848-2424, extension 8658 or by email at the following address: [ombuds@concordia.ca](mailto:ombuds@concordia.ca)

**17. Monitoring of ethical aspects of the research project**

The Comité central d'éthique de la recherche du ministre de la santé et des services sociaux has approved the research project and will monitor it. For any information, you can contact Ms. Johane de Champlain, vice-president.

**Consent**

| **Title of research project:** | Strengthening and Targeting Rehabilitation for Optimal Neuromuscular Gains for chronic BACK pain (STRONG-BACK): A randomized controlled trial in participants with primary nociceptive pain drivers. |
| --- | --- |

1. **Participant’s Consent**

I have read the information and consent form. I recognize that the project was explained to me, that all my questions were answered and that I was given sufficient time to make a decision.

Upon reflection, I agree to participate in this research project under the conditions that are described.

Signature du participant Date

1. **Signature of person that obtained the consent if different from the researcher responsible for the research project.**

I explained to the participants the terms of this Information and Consent Form and I answered his questions.

Signature of the person that obtained consent Date

1. **Signature and engagement of the researcher responsible for the research project**

I certify that we explained to the participant the terms of this information and consent form, answered all the questions he had in this regard and have clearly indicated that he remains free to terminate its participation without prejudice.

I agree with the research team, to respect what was agreed at the information and consent form and give a copy signed and dated to the participant.

Signature of the researcher responsible for the research project Date
